# Transcriptomic Study of Porcine Small Intestine Epithelial Cells Reveals Important Genes and Pathways Associated With Susceptibility to *Escherichia coli* F4ac Diarrhea

**DOI:** 10.3389/fgene.2020.00068

**Published:** 2020-02-27

**Authors:** Serafino M. A. Augustino, Qinglei Xu, Xueqin Liu, Lei Liu, Qin Zhang, Ying Yu

**Affiliations:** ^1^ Key Laboratory of Animal Genetics, Breeding and Reproduction, Ministry of Agriculture & National Engineering Laboratory for Animal Breeding, College of Animal Science and Technology, China Agricultural University, Beijing, China; ^2^ Research Centre for Animal Genomic, Agricultural Genomic Institute at Shenzhen, Chinese Academy of Agricultural Sciences, Shenzhen, China

**Keywords:** diarrhea, ETEC F4ac fimbriae, intestine epithelial cells adhesion, piglet's susceptibility phenotype, *RNA-Seq*

## Abstract

**Background:**

Diarrhea represents one of the most frequent major problems during piglets' neonatal and post-weaning periods leading to tremendous economic losses in the swine industry. Enterotoxigenic *Escherichia coli* (ETEC) F4 is regarded as the most important cause of diarrhea in piglets. However, some pigs are naturally resistant to those diarrheas caused by ETEC-F4, because they have no F4 receptors (F4R) on their small intestine epithelial cells that allow F4 fimbriae attachment. Thus, our study characterized a complete transcriptome of small intestine epithelial cells of Large White piglets using RNA-Seq. The aim of the study was to identify DEGs with regard to differences in the F4R phenotypes and SNP (C/T) genotypes at *ITGB5* and important pathways associated with ETEC-F4ac susceptibility in small intestine epithelial cells of Large White piglets and derive molecular markers as a result of loss of F4acR in swine.

**Methods:**

A total of eight samples of small intestine epithelial cells obtained from Large White piglets (35 days old) used in this study were selected on the basis of two criteria. One was the adhesion phenotype to ETEC-F4ac fimbriae, and the other was the comparison of *ITGB5* SNP (C > T) genotype sequences across all the samples. The samples were then divided into two groups, non-adhesive with CC genotype (n = 4), and adhesive with TT genotype (n = 4).

**Results:**

More down-regulated DEGs (p < 0.05, |log_2_FC| > 2) were detected in the comparison of non-adhesive vs. adhesive small intestine epithelial cells in the present study. Six genes, of which two (*CNGA4, SLC25A31*) exclusively expressed and four (*HCN4, MYLK, KCNMA1*, and *KCNMB1)* DEGs with up-regulation pattern in adhesive (F4R positive) pigs were involved in two pathways associated with diarrhea. The DEGs with up-regulation pattern in non-adhesive (F4R negative) pigs were mostly engaged in multiple immune response-related pathways.

**Conclusion:**

The results provide insights on the biology of the phenotypes of F4R positive and negative pigs. One gene (*MYLK*) located on SSC13 locus for F4acR strongly support that it might have played a role in the adhesion phenotype which was obviously detected by adhesion assay in adhesive (F4R positive) group.

## Introduction

Diarrhea can best be defined as the passage of watery or loose stools, always with frequent movements of bowel owing to infection (Schiller et al., 2014). Enterotoxigenic *Escherichia coli* expressing F4 fimbriae (ETEC-F4) associated diarrhea results in morbidity and mortality in neonatal and post-weaned piglets. This disease is accountable for 11.5%–29.5% of piglets' deaths worldwide and is considered as one of the economically most important diseases in pig farms (Rasschaert et al., 2010; Nguyen et al., 2017). Improvement of the environment and application of preventive measures such as vaccination of the sows can only reduce the incidence rate of diarrhea. Susceptibility to ETEC-F4 is dominantly inherited following Mendelian inheritance (Ren et al., 2012; Liu et al., 2017). Previous studies proved that some pigs are naturally resistant to ETEC-F4 diarrheal infection, because they do not have F4 receptors (F4R) on their epithelial cell brush borders that to which the fimbriae adhere, i.e., they are non-adhesive pigs or resistant pigs (Baker et al., 1997; Geenen et al., 2004). Therefore, if measures that lead to the improvement of piglets' resistance level to diarrhea from the genetic side are followed, can extremely reduce the incidence of diarrhea in piglets, improve the safety of pork products and consequently boost the economy of swine industries.

The molecular mechanisms underlying susceptibility to diarrhea caused by ETEC-F4 in piglets remain largely unclear. Fu et al. (2012) explored potential causal genes for F4R in three pig breeds (including Large White, Landrace and Songliao Hei) using genome-wide association study (GWAS) and found 18 significant SNPs with known locations in the porcine genome associated with resistance to ETEC-F4. However, our understanding of the research to date is that there may be several different receptors (and genes) involved at the resistance locus on SSC13 in part due to the variation in *E coli* F4. A transcriptional approach may help “dissect” what is going on, but in terms of the receptor (F4R), an analysis of gene expression of the genes in the region would be a key aspect of such an effort.

The expression level of genes transcripts represents the most immediate phenotype that can be associated with disease resistance or susceptibility (Wang et al., 2010; do Nascimento et al., 2018).

Nowadays, high-throughput RNA sequencing (RNA-seq) technique has become a powerful tool and a standard method for the measurement and comparison of genes expression levels (Pertea et al., 2016). One of the most biologically relevant applications of RNA-Seq is the comparison of mRNA transcriptomes between infected and healthy individuals (Mirhashemi et al., 2018; Barroso and McCarthy, 2019). The Large White pig is a domestic pig breed originating in Yorkshire in the UK and currently is mostly reared commercial pig worldwide. In this study, we characterized a complete transcriptome of non-adhesive vs. adhesive small intestine epithelial cells of Large White piglets using RNA-seq techniques and bioinformatic analyses. This study aimed mainly at 1. Identifying genes exclusively and differentially expressed in the small intestine epithelial cells of large white piglets differing in ETEC F4ac adhesion phenotypes and SNP (C > T) genotypes at *ITGB5*. 2. Predicting pathways associated with diarrhea susceptibility from the analysis of the DEGs 3. Exploring the potential molecular markers as a result of absence and presence of F4acR expression in small intestine epithelial cells.

## Material and Methods

### Animals Management and Samples Collection

The experiment was conducted in accordance with the protocol approved by the Animal Welfare Committee of China Agricultural University (Permit Number: DK996). The experimental animals used in this study were all male Large White Piglets, which were raised under standard indoor conditions at the experimental farm of the Institute of Animal Sciences, Chinese Academy of Agricultural Sciences. At 35 days of age, piglets were slaughtered, small intestine tissue samples were obtained from Large White piglets within 30 min after slaughter. Each tissue sample was cut along its longitudinal axis, washed with a hypotonic EDTA solution (5 mmol/L EDTA, pH 7.4), placed in a liquid nitrogen container, transferred immediately to the laboratory and stored at –80°C until used for adhesion assay. For more detail on animal population and sampling, and slaughtering procedures etc. please refer to (Li et al., 2007).

### Adhesion Assay

Adhesion herein, refers to the process or tendency of ETEC-F4 fimbriae to adhere to intestinal epithelial cells F4 receptors in susceptible piglets. The susceptibility phenotype of small intestine of Large White piglets to ETEC-F4ac strain 200 (C83907, O149:K91) was performed using *in vitro* adhesion test. This strain was provided by the China Institute of Veterinary Drug Control, Beijing, China.

The adhesion assay was completed by research group in our lab, more details on the procedures of *in vitro* adhesion test and the results of the adhesion assay can be found in our previous study (Li et al., 2007).

Briefly, the bacterial suspension and the brush border cell suspension (0.1 ml each) were mixed with 0.4 mg/ml mannose then put in the incubation for 30 min at room temperature. Subsequently, a drop of the suspension mixture was checked for adhesion using a phase-contrast microscopy. A single epithelial cell was considered to be adhesive when there were more than five bacteria adhering to the brush border membrane. Over 20 epithelial cells from the epithelial cell specimen of a piglet were checked and the piglet was regarded as strongly adhesive when at least 80% of the epithelial cells were judged as adhesive. Adhesive when 10% to 80% of the epithelial cells were adhesive. Weakly adhesive when less than 10% of the epithelial cells were adhesive, or non-adhesive when no epithelial cells were adhesive. The terms non-adhesive (small intestine epithelial cells without F4R) and adhesive (small intestine epithelial cells with F4R) will be used interchangeably with resistant and susceptible respectively, in the text. Because, it is widely accepted that resistance and susceptibility of small intestine epithelial cells to diarrhea induced by ETEC-F4 is based on the absence and presence of F4R, respectively.

### Genotyping of Piglets

After adhesion assay, for determining the genotypes of the piglets with F4R (adhesive) and without F4R (non-adhesive) on their small intestine epithelial cells, we referred to our previous study (Xu et al., 2019) which sequenced the genotypes of these piglets using integrin subunit beta 5 (*ITGB5)* SNP NC_010455.5 (g.135577826 C > T) which was identified by GWAS study in our lab (Fu et al., 2012).

### Experimental Animals

After adhesion assay and genotyping, eight (8) piglets were selected on the basis of two criteria, adhesion phenotype (adhesive and non-adhesive small intestine epithelial cells) and *ITGB5* SNP (C > T) genotype. They were then divided into two groups. Adhesive small intestine epithelial cells (adhesive group) with TT genotype (n = 4) and non-adhesive small intestine epithelial cells (non-adhesive group) with CC genotype (n = 4).

### Total RNA Extraction and Quality Examination

The TRIzol reagent (Invitrogen, Carlsbad, CA) was used to isolate the total RNA from each sample of Large White piglet's small intestine epithelial cells with adhesive and non-adhesive phenotypes following the manufacturer's protocol. The concentrations of the isolated RNA were determined using the Nano Drop spectrophotometer. The purity of RNA was checked using the kaiaoK5500^®^Spectrophotometer (Kaiao, Beijing, China). The degradation and contamination of RNA quality was checked on 1% agarose gels revealed three distinct bands of 28S, 18S, and 5S (**Supplementary Figure 1**). RNA integrity and concentration was assessed using the RNA Nano 6000 Assay Kit of the Bioanalyzer 2100 system (Agilent Technologies, CA, USA). The quality of all the RNA samples were good enough (OD260/280 > 1.90, RNA integrity number > 8.7) to do the sequencing. Then 20 µL of the isolated total RNA from each sample were sent to company (Annoroad Gene Technology Corporation -Beijing) for sequencing. The rest were stored at –80°C until used for the validation of some genes by RT-qPCR.

### Library Preparation for RNA Sequencing

A total amount of 2 μg RNA per sample was used as an input material for the RNA sample preparations. Sequencing libraries were generated using NEBNext^®^ Ultra™ RNA Library Prep Kit for Illumina^®^ (#E7530L, NEB, USA) following the manufacturer's recommendations and index codes were added to attribute sequences to each sample. Briefly, mRNA was purified from total RNA using poly-T oligo-attached magnetic beads. Fragmentation was carried out using divalent cations under elevated temperature in NEBNext First Strand Synthesis Reaction Buffer (5X). First strand cDNA was synthesized using random hexamer primer and RNase H. Second strand cDNA synthesis was subsequently performed using buffer, dNTPs, DNA polymerase I, and RNase H. The library fragments were purified with QiaQuick PCR kits and elution with EB buffer, then terminal repair, A-tailing and adapter added were implemented. The aimed products were retrieved and PCR was performed, then the library was completed.

RNA concentration of library was measured using Qubit^®^ RNA Assay Kit in Qubit^®^ 3.0 to preliminary quantify and then dilute to 1 ng/μl. Insert size was assessed using the Agilent Bioanalyzer 2100 system (Agilent Technologies, CA, USA), and qualified insert size was accurate quantification using StepOnePlus™ Real-Time PCR System (Library valid concentration >10 nM). The clustering of the index-coded samples was performed on a cBot cluster generation system using HiSeq PE Cluster Kit v4-cBot-HS (Illumina) according to the manufacturer's instructions. After cluster generation, the libraries were sequenced on an Illumina platform (HiSeq Xten) and 150 bp paired-end reads were generated.

### RNA-Seq Data Analysis

After RNA sequencing, no pre-processing or upstream analysis with regard to quality control was performed as we obtained clean reads from the sequencing company. Briefly, the clean data (clean reads) were obtained by removing reads containing adapters, reads containing more than 5% Ns, reads with Qphred ≤ 19, and low quality reads from raw reads. RNA-Seq paired-end clean data were then analyzed using Hisat2-2.1.0. and samtools-1.9, Subread 1.6.3 (Liao et al., 2019) and DESeq2 1.10.1 (Love et al., 2014). Porcine reference genome (Susscrofa 11.1) was obtained from Ensembl (ftp://ftp.ensembl.org/pub/release-96/fasta/sus_scrofa/dna/). Then, the index of reference genome was created by build-index function in Hisat2-2.1.0 software (http://ccb.jhu.edu/software/hisat2) package with default options. Hisat2 was then employed to align paired-end clean reads of each sample to the reference genome with default settings. The generated SAM files were converted to BAM files using samtools-1.9 software (http://samtools.sourceforge.net). Subsequently, FeatureCounts function in Subread 1.6.3 was employed to generate raw counts with the BAM files. Finally, the generated raw counts were used as inputs in DESeq2 employed in R version (3.5.2). We left genes with read counts greater than 10 and have read counts in at least two samples for differential expression analysis with DESeq2.

Differential expressed genes (DEGs) analysis was performed using DESeq2 to determine DEGs in comparison of non-adhesive vs. adhesive small intestine epithelial cells (P value < 0.05, log2 |FC| > 1). Raw sequencing data for eight samples analyzed in this study were submitted to the National Center for Biotechnology Information (NCBI), Sequence Read Archive (SRA) and are available under accession number: PRJNA562774.

### Gene Ontology (GO) Annotation and KEGG Analysis

Gene ontology (GO) enrichment and KEGG pathway analyses were performed using a web based software called Database for Annotation, Visualization and Integrated Discovery (DAVID) version 6.8 (https://david.ncifcrf.gov/content.jsp?file=DAVID_Publications.html.n.d). Species *Sus scrofa* was used as background.

### Protein-Protein Interaction Network Analysis

Search Tool for the Retrieval of Interacting Genes/proteins (STRING), a web based tool, was used to reveal protein-protein interaction network of the genes using organism *Sus scrofa* as background (https://string-db.org/cgi/input.pl)n.d).

### Complementary DNA (cDNA) Synthesis for RT-qPCR

Complementary DNA (cDNA) was synthesized from total RNA using PrimeScriptTM RT reagent kit with gDNA Eraser (Perfect Real Time) (TAKARA BIO INC.) following manufacturer's instructions. Reverse transcription reactions were performed in a final volume of 20 µl using the following PCR reaction conditions 37°C for 15 min and 85°C for 5 sec and stored at –20°C for RT-qPCR later.

### Quantitative Real-Time -PCR (RT-qPCR)

RT-qPCR reactions were performed in a final volume of 20 µl with the Roche SYBR Green PCR Kit (Roche, Hercules, CA, USA) using a Light-CyclerH 480 Real-Time PCR System (Roche, Hercules, CA, USA) to validate several RNA-Seq DEGs.

The porcine reference gene glyceraldehyde-3-phosphate dehydrogenase (*GAPDH)* was used as the internal standards to adjust the input of cDNA and to normalize the expression of the selected genes. Duplicate RT-qPCRs were conducted on each cDNA sample and the average Ct was used for the analysis. Briefly, the difference in cycle times, ΔCt, was determined as the difference between the test gene and the reference housekeeping gene. The ΔΔCt was obtained by finding the difference between samples. The relative mRNA expression levels of the 16 genes were analyzed against swine housekeeping gene using the 2^−△△Ct^ method. The primers used to amplify the mRNAs of these genes were designed using Primer3 online software (https://www.ncbi.nlm.nih.gov/tools/primer-blast/index.cgi?) and were further checked for primer dimer and primer self-complementarity using oligo6 software Version 6.41. The primers used for RT-qPCR validation are listed in (**Table 1**). The cycling reaction conditions were as follows: one cycle of 95°C for 5 min, 45 cycles of 95°C for 20 sec, 58°C for 30 sec, and 68°C for 45 sec, and one cycle of 72°C for 10 min. A single peak melting curve was used to validate the specificity of the RT-qPCR amplification.

**Table 1:** Primers for qPCR validation of differential expressed genes (DEGs) in the comparison of non-adhesive vs. adhesive small intestine epithelial cells in Large White piglets.

DEGs	Primer’s sequence 5’ – 3’	Tm	Product size
*DDX3X*	F ACAGCAGTTTTGGATCCCGTA R CACTTCGTCCACGGTCATCA	59.65 60.04	102
*ARPC4*	F TCGACAAGGAGATCAGTGAGA R CAGACACAGGGAGCTTGAGG	57.93 60.04	132
*CXCL2*	F CAGCCCCCATGGTGAAGAAA R AGCAGTAGCCAGTAAGTTTCCTC	60.25 60.06	94
*CYCS*	F CTGCGAGTGGTGGCTTGT R TGCCCTTCTCAACATCACCC	60.28 59.96	71
*CTLA4*	F GCAGCAGTTAGTTCAGGGTTG R GTCGGGGGCATTTTCACATAG	59.46 59.32	119
*IL1R2*	F ACCATGAGTTTCCAGACGCTT R AAAACCAGGATGATGAGGGCG	59.93 60.68	98
*MGAT2*	F CGGGCCGAATACCTCAAACT R TGGCTAAAGATGACGAGGACG	60.11 59.87	80
*RBP2*	F AAAGGGGAAAAGGAGAACCGAG R GAACACTTGACGGCACACCTG	60.22 61.99	99
*SLC22A8*	F ACGGCATAGCAGACCTGTTC R CCTCCACACCCATAGCCAAA	60.11 59.67	114
*RUNX1T1*	F TGGAATTGTGGCCGTAAAGC R GGTGCTTCTCCCAGTCTTTGT	59.11 60.20	97
*ACTN2*	F TTCATGACCAGGGAGACAGC R TCTCGACGCAGCTCCTCT	59.38 60.05	110
*CACNB2*	F TCCCCAACCCTCTCCTCAG R CGGGCAGGCTTCTTCTTCA	59.92 60.00	155
*CLDN11*	F TACACTATTCCCACCTGCCG R GACAGAGGCAGCGATCATCA	60.20 60.74	165
*DGKG*	F TGGAACCGGCAATGACCTG R CCAGCATCACCAAGGGACTC	60.30 60.39	110
*HCN4*	F CCATGCTGCAGGACTTCCC R GCCTTGAAGAGGGCGTAGG	60.75 60.15	97
*NCAM1*	F GAGAACCAGCAGGGGAAGTC R AAACGTGTAGGACGCGGAG	60.04 60.08	108
*GAPDH*	F :CCACGGTCCATGCCATCACT R :GCCTGCTTCACCACCTTCTTG		268

## Results

### Genotyping of Piglets’ Small Intestine With Adhesive and Non-Adhesive Phenotype

**Figure 1:**
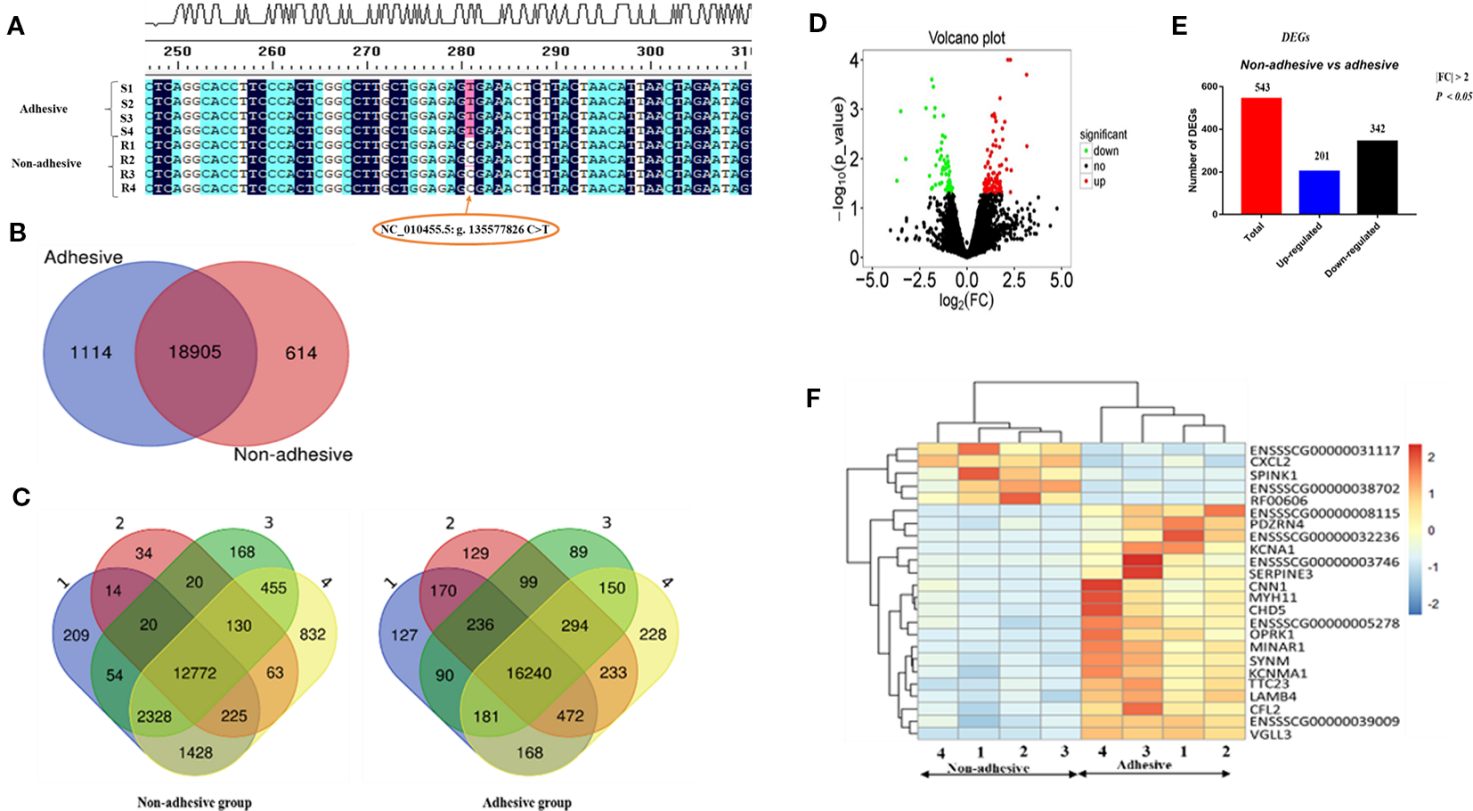
Porcine *ITGB5* genotyping and differential expressed genes (DEGs) in the comparison of non-adhesive vs. adhesive small intestine epithelial cells of Large White piglets: **(A)** Classification of the eight samples with adhesion and non-adhesion of ETEC-F4ac using *ITGB5* SNPs sequences results. R stands for non-adhesive (resistant), S stands for adhesive (susceptible) phenotypes. Allele C associated with resistance and allele T associates with susceptibility (Fu et al., 2012) **(B)** Venn diagram showing genes only expressed in non-adhesive small intestine epithelial cells (red circle), genes only expressed in the adhesive small intestine epithelial cells (blue circle), and genes in intersection are common to both groups. **(C)** Number of genes and overlapping genes in non-adhesive small intestine and adhesive samples epithelial cells. **(D)** Volcano plot: showing significantly up-regulated DEGs (red dots), significantly down-regulated DEGs (green dots), and non-significantly DEGs (black dots). **(E)** Total number of DEGs (red bar), up-regulated (blue bar), and down-regulated (black bar) in comparison of non-adhesive vs. adhesive small intestine epithelial cells. **(F)** Heatmap of genes expression levels for the top 25 (DEGs) in the comparison of adhesion and non-adhesion small intestine epithelial cells.

The adhesive and non-adhesive small intestine epithelial cells phenotypes in Large White piglets were significantly associated with the genotypes of integrin subunit beta 5 (*ITGB5)* SNP NC_010455.5 (g.135577826 C > T) (Fu et al., 2012). It means *ITGB5* could be a promising candidate gene for F4R to which ETEC-F4ac binds. For determining the genotypes of the gene in the eight samples, we sequenced the genotypes of the SNP in *ITGB5* (Xu et al., 2019) for all the samples and compared them as shown in **Figure 1A**. We observed that all samples of adhesive small intestine epithelial cells were TT genotypes, while all samples of non-adhesive small intestine epithelial cells were CC genotypes, which were consistent with our previous genome wide association study (Fu et al., 2012).

### Transcriptome Profiling of Adhesive and Non-Adhesive Small Intestine Epithelial Cells in Large White Piglets

To profile the transcriptome of Large White piglets' small intestine epithelial cells with adhesive and non-adhesive phenotype, we performed a complete transcriptomic analysis using RNA-seq techniques (**Supplementary Tables S1** and **S2**). Out of the 25,880 pig annotated genes (Sscrofa 11.1) in the Ensembl database (asia.ensembl.org/biomart/martview), 20,346 genes were found expressed in the samples. Among these genes, 20019 and 19519 were expressed in small intestine epithelial cells with adhesive and non-adhesive phenotype, respectively, (**Table 2**). Furthermore, we identified 1,114 and 614 genes that were exclusively expressed in adhesive and non-adhesive small intestine epithelial cells group, respectively, (**Figure 1B**, **Table 2**) with 18,905 shared genes between the two groups (**Table 2**).

**Table 2:** Summary of RNA-Seq analysis results.

Total Genes			Expressed genes			Exclusively expressed genes		DEGs (Non-adhesive vs adhesive) (*P value < 0.05,* log2|FC| > 1)		
Ensembl data base	N/A	Expressed genes	Adhesive	Shared	Non-adhesive	Adhesive	Non-adhesive	Total	Up-regulated	Down-regulated
25880	5523	20,346	20,019	18,905	19,519	1,114	614	543	201	342

Samples of small intestine epithelial cells within non-adhesive group shared less genes in common (*n* = 12,772) as compared to that of small intestine epithelial cells within adhesive group (*n* = 16,240) (**Figure 1C**). A total of 822 DEGs (*P < 0.05,* log2 |FC| > 0) were found in the comparison of small intestine epithelial cells in non-adhesive vs. adhesive group (**Supplementary Table S3**). In addition, using criteria *P < 0.05* and log2 |FC| > 1, we observed that the number of down-regulated DEGs (342) was more than up-regulated DEGs (201) in the comparison of small intestine epithelial cells in non-adhesive vs. adhesive group (**Figures 1D, E**). The heatmap of the top 25 DEGs revealed 5 genes were clustered as up-regulated in small intestine epithelial cells in non-adhesive group and 20 genes were clustered as up-regulated in small intestine epithelial cells in adhesive group (**Figure 1F**). These results indicated that significant transcriptional profiles were observed between the two groups with different F4R phenotypes and *ITGB5* genotypes.

### Functional Enrichment Analyses

**Figure 2:**
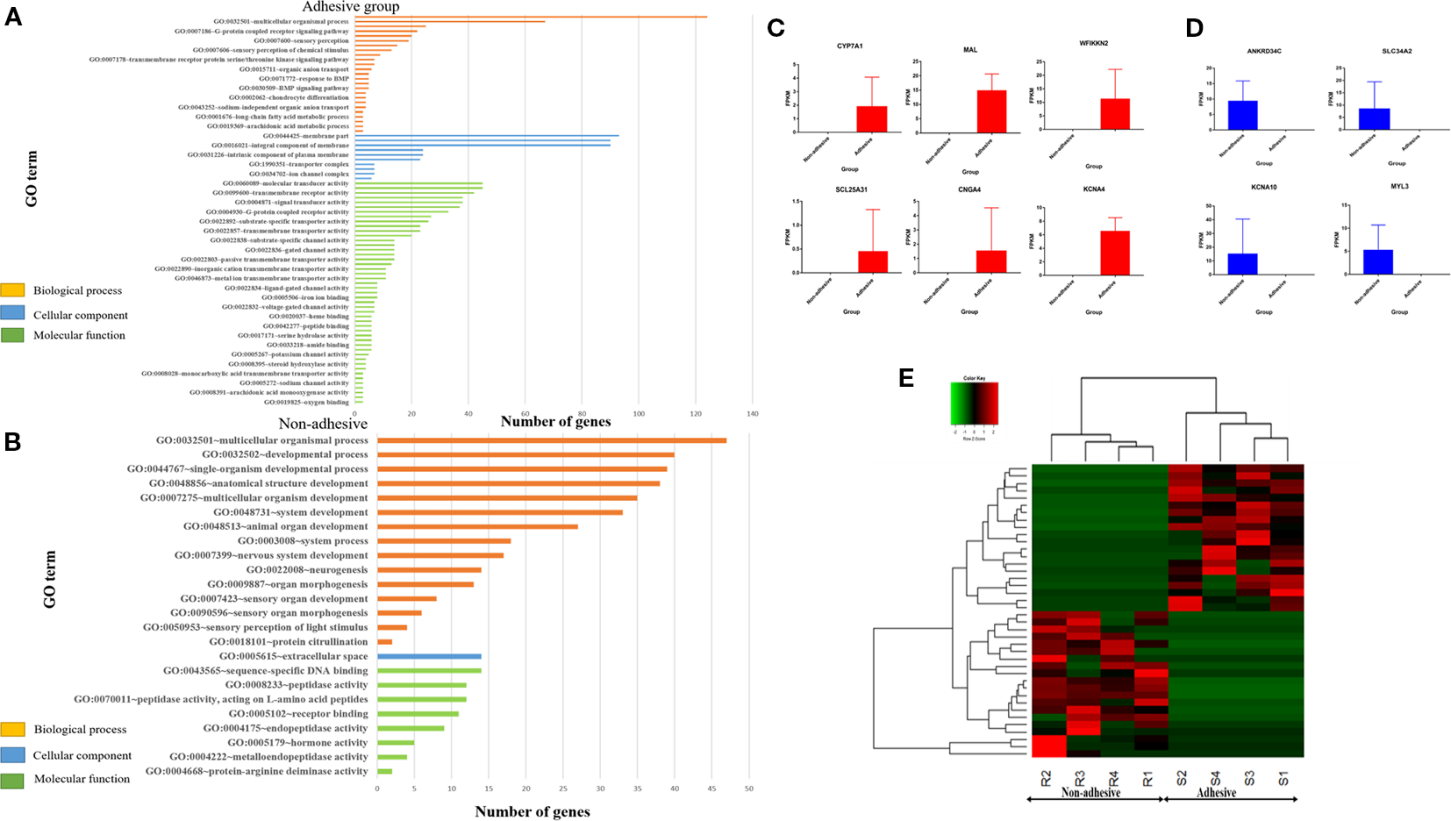
Significantly enriched (p < 0.05) GO term of genes exclusively expressed in adhesive and non-adhesive group: Biological process, cellular component and molecular function of genes exclusively expressed in adhesive group **(A)** and non-adhesive group **(B)**. Graph showing some of exclusively expressed genes in small intestine epithelial cells in adhesive group **(C)** and non-adhesive group **(D)** using FPKM values. **(E)** Heatmap of top 20 genes exclusively expressed in adhesive and non-adhesive epithelial cells.

**Figure 3:**
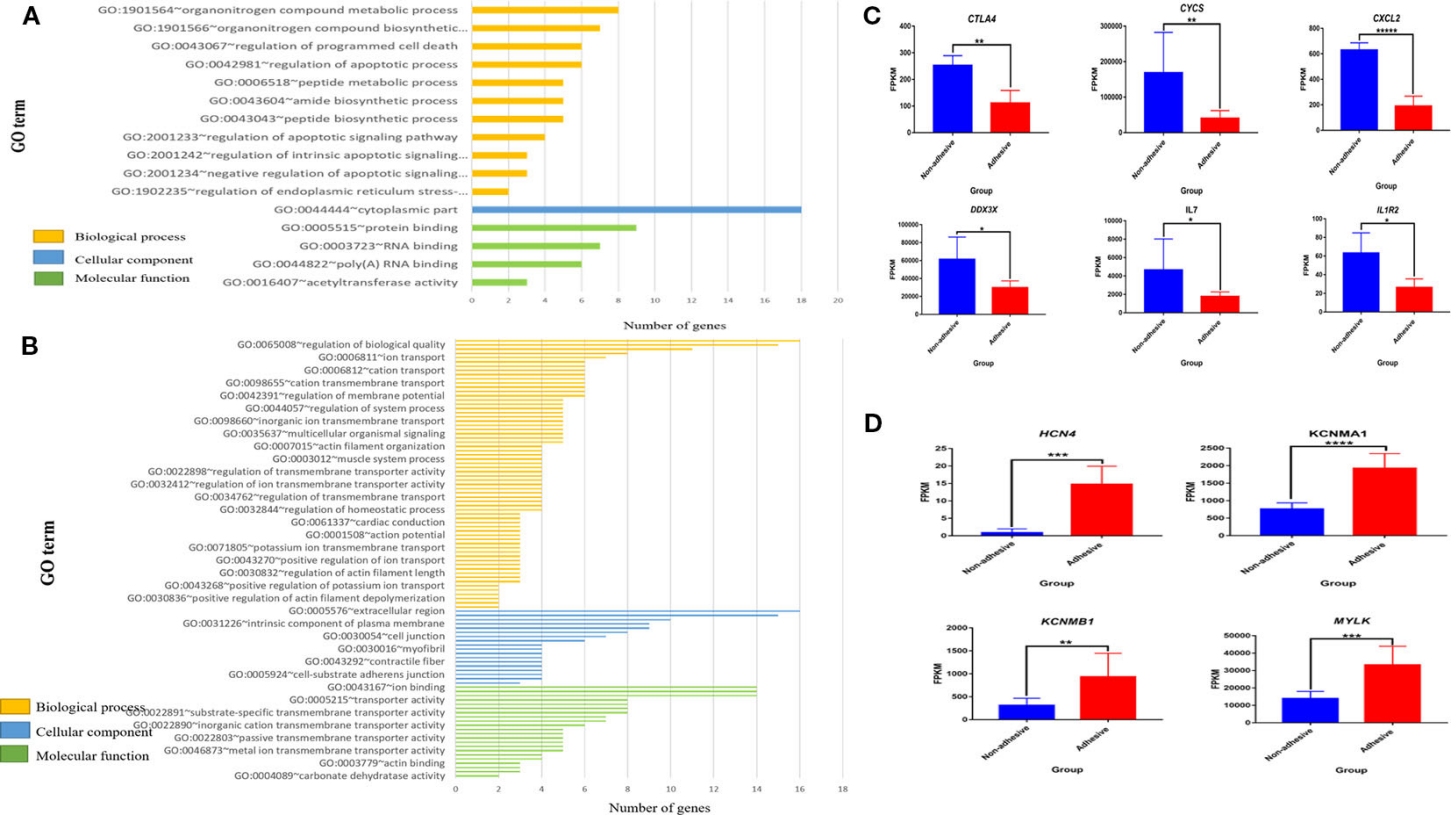
Significantly enriched (p < 0.05) GO term of differential expressed genes (DEGs) and differential expression of key genes in comparison of non-adhesive vs adhesive small intestine of large white piglets: **(A)** Biological process, cellular component and molecular function of the top 100 up-regulated DEGs. **(B)** Biological process, cellular component and molecular function of the top 100 down-regulated DEGs. **(C)** Down-regulated differential expressed genes associated with diarrhea susceptibility in adhesive small intestine epithelial cells using FPKM values **(D)** Up-regulated differential expressed genes related to immunity in non-adhesive small intestine epithelial cells using FPKM values.

To identify genes that are involved in important biological processes and pathways, Gene Ontology and KEGG were performed for all genes exclusively expressed in small intestine epithelial cells in adhesive and non-adhesive group and DEGs in comparison of small intestine epithelial cells in non-adhesive vs. adhesive group using DAVID version 6.8, a web based software. Only the significantly enriched (P < 0.05) GO terms are listed in (**Figures 2A, B**, **Figures 3A**, **B**).

Gene Ontology analysis of genes exclusively expressed in small intestine epithelial cells in adhesive group revealed 25 signiﬁcantly enriched biological processes, 10 significantly enriched cellular components, and 47 significantly enriched molecular functions with *P < 0.05 *(**Figure 2A**). Whereas genes exclusive expressed in small intestine epithelial cells in non-adhesive group revealed 15 significantly enriched biological processes, one significantly enriched cellular component and eight significantly enriched molecular functions with *P < 0.05* (**Figure 3B**). Genes exclusively expressed in small intestine epithelial cells in adhesive group were mostly engage in single-organism process, multicellular organismal process, cellular response to endogenous stimulus, and epipoxygenase P450 pathway (**Figure 2A**). Whereas those genes exclusively expressed in small intestine epithelial cells in non-adhesive group were mostly involved in multicellular organismal process, developmental process, and single-organism developmental process (**Figure 2B**).

KEGG analysis of genes exclusively expressed in small intestine epithelial cells in non-adhesive group revealed one significantly enriched pathways (**Supplementary Table S4**) which is shared with non-adhesive group. Whereas genes exclusively expressed in small intestine epithelial cells in adhesive group revealed six significantly enriched pathway (**Supplementary Table S5**). Two genes (*CNGA4, SLC25A31*) (**Figure 2C**) exclusively expressed in adhesive small intestine epithelial cells were involved in cAMP and cGMP pathway respectively (**Supplementary Table S6**). Some of genes exclusively expressed in non-adhesive group are shown in **Figure 2D**.

Gene Ontology analysis of 100 top up-regulated and 100 top down-regulated DEGs in the comparison of small intestine epithelial cells in non-adhesion vs adhesion group revealed 11 signiﬁcantly enriched biological processes in non-adhesive small intestine epithelial cells with up-regulated DEGs (**Figure 3A**, **Supplementary Table S7**) compared to 109 significantly enriched biological processes in the adhesive small intestine epithelial cells with down-regulated DEGs (**Figure 3B**, **Supplementary Table S8**). Only one cellular component (**Figure 3A**, **Supplementary Table S9**) was found significantly enriched in non-adhesive group with up-regulated DEGs in comparison to 28 significantly enriched cellular components (**Figure 3B**, **Supplementary Table S10**) in adhesive group with down-regulated DEGs.

Furthermore, four significantly enriched molecular functions in non-adhesive group with up-regulated DEGs (**Figure 3A**, **Supplementary Table S11**), compared to 25 in adhesive group with down-regulated DEGs (**Figure 3B**, **Supplementary Table S12**). None of the significantly enriched biological processes was found shared between up- and down-regulated DEGs.

Most significantly enriched biological processes in small intestine epithelial cells in non-adhesive group with up-regulated DEGs were mainly apoptotic related biological processes (**Figure 3A** and **Supplementary Table S7**). The up-regulation of DEGs could have resulted as a result of a lack of F4R in non-adhesive small intestine epithelial cells. While adhesive small intestine epithelial cells with down-regulated DEGs mostly involved in channels and ions transmembrane transporter activities (**Figure 3B** and **Supplementary Table S8**). This suggests that, the presence of a functional F4R is behind the up-regulation of DEGs in adhesive group.

To reveal the important biological pathways in which DEGs are involved, we performed KEGG analysis for the top 100 up-regulated and top 100 down-regulated DEGs. The results revealed three up-regulated genes (*SEC22B, CXCL2*, and *CYCS*) and two down-regulated genes (*CA3, CA4*) were significantly involved in Legionellosis and nitrogen metabolism pathways, respectively (**Table 3**). Three pathways namely Adhesion junction (ssc04520), Cell adhesion molecules (ssc04514) and Metabolic pathways (ssc01100) were shared between up- and down-regulated DEGs in both non-adhesive and adhesive small intestine epithelial cells (**Supplementary Tables S13** and **S14**).

**Table 3:** Key genes and important KEGG pathways involved in ETEC-F4ac non-adhesion and adhesion.

	KEGG pathway	KEGG ID	DEGs	P value
Up-regulated	Legionellosis	ssc05134	*SEC22B, CXCL2, CYCS*	1.5E-2
	Cytokines cytokine receptor interaction	ssc04060	*IL1R2, IL7*	–
	Chemokine signaling pathway	ssc04060	*CXCL2*	–
	TNF signaling pathway	ssc04668	*CXCL2*	–
	T cell receptor signaling pathway	ssc05660	*CTLA4*	–
	Autoimmune thyroid disease	ssc05320	*CTLA4*	–
	Cell adhesion molecules	Ssc04514	*CTLA4*	–
	p53 signaling pathway	ssc04115	*CYCS*	–
Down-regulated	Nitrogen metabolism	ssc00910	*CA3, CA4*	3.4E-2
	cAMP signaling pathway,	ssc04024	*HCN4*	–
	cGMP-PKG signaling pathways	ssc04022	*MYLK, KCNMB1, KCNMA1*	–
	Cell adhesion molecules	ssc04514	*NCAM1, CLDN11*	–
	Focal adhesion	ssc04510	*ACTN2, MYLK*	–
	MAPK signaling pathway	Ssc04010	*CACNB2*	–

In the comparison of non-adhesive vs adhesive small intestine epithelial cells, some important DEGs were observed, of which six (*CXCl2, CYCS, CTLA4, IL1R2, IL7*, *and DDX3X*) are immune-related genes (**Supplementary Table S13**). All of them were up-regulated in non-adhesive than in adhesive small intestine epithelial cells (**Figure 3C**). This explains that, the absence of a functional F4R in small intestine epithelial cells might also have resulted in their overexpression. Whereas four DEGs (*HCN4, KCNMA1, KCNMB1*, *and MYLK*) (**Supplementary Table S14**) were associated with pathways related to ETEC-F4ac diarrhea susceptibility and were all up-regulated in small intestine epithelial cells in adhesive group than in non-adhesive group (**Figure 3D**). This indicates that, the overexpression of these genes may be due to the presence of a functional F4R that permits adhesion of ETEC-F4ac to small intestine epithelial cells which was obviously detected by adhesion assay in the adhesive (F4R positive) group.

Interestingly *DDX3X* (ENSSSCG00000012252) in **Figure 3C** was found involved in all significantly enriched biological processes in small intestine epithelial cells in non-adhesive group with up regulation pattern, except one, GO:1902235~regulation of endoplasmic reticulum stress-induced intrinsic apoptotic signaling pathway (**Supplementary Table S7**). This suggests that, it has critical roles to play in small intestine epithelial cells in non-adhesive group as an important biological molecule. Its involvement in ssc04622: RIG-I-like receptor signaling pathway, ssc05161: Hepatitis B and ssc05203: Viral carcinogenesis (**Supplementary Table S10**) could also suggest it plays an important role in innate immunity in non-adhesive (F4R negative) group.

### Protein-Protein Interaction (PPI) Analysis of the DEGs in the Comparison of Non-Adhesion vs Adhesion

**Figure 4:**
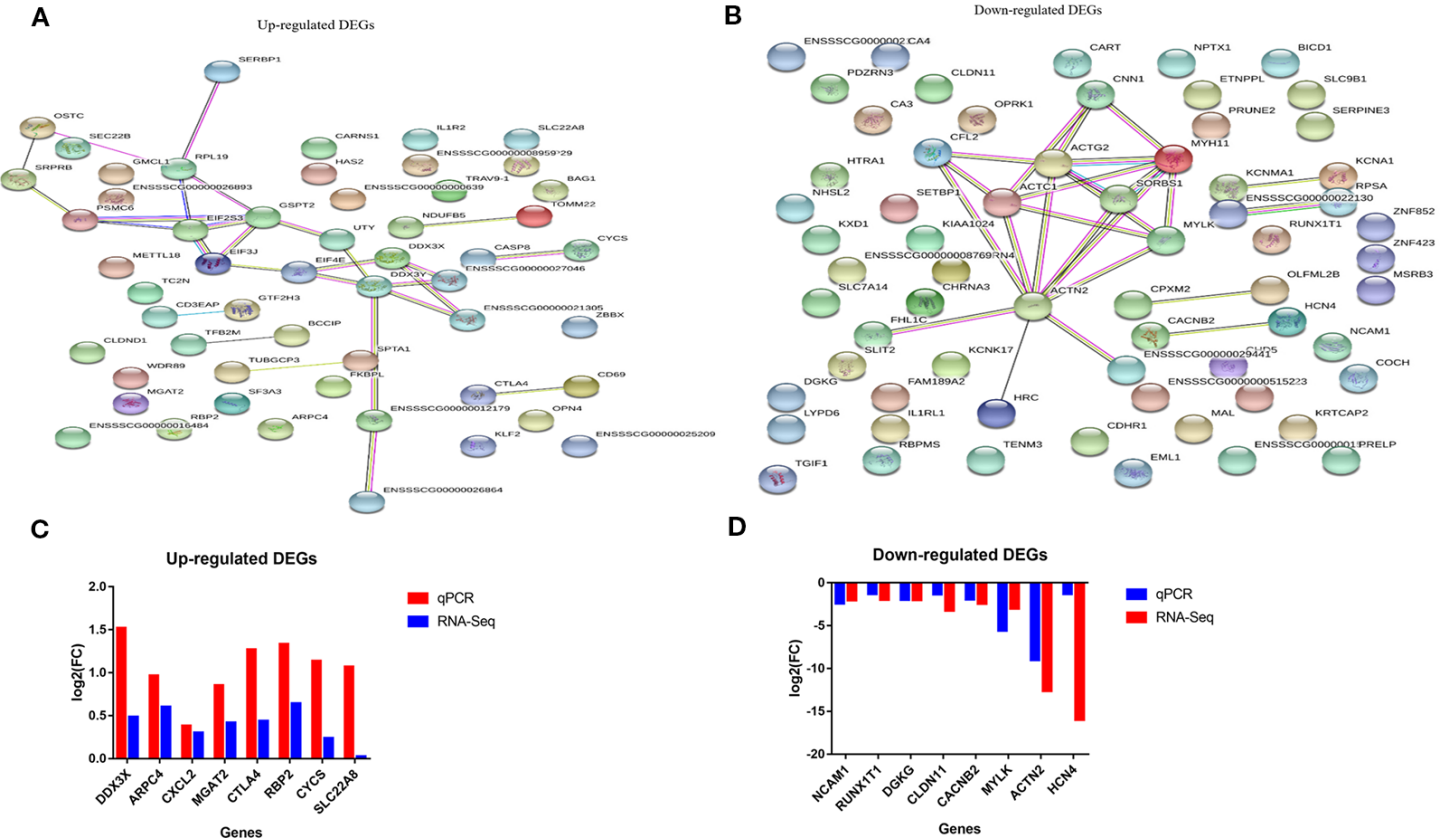
String analysis and qRT-PCR validation of the differential expressed genes (DEGs) **(A, B)** Protein-protein interaction network of up-and down-regulated DEGs in comparison of non-adhesive vs adhesive small intestine of large white piglets respectively. **(C, D)** Validation of up- and down-regulated DEGs with qRT-PCR respectively in comparison of non-adhesive vs adhesive small intestine of large white piglets.

In order to gain insights into deep understanding of the relationships between DEGs biologically, PPI networks were constructed using the 100 top up-regulated and 100 top down-regulated DEGs in STRING (a web based tool: https://string-db.org). Of the 100 up-regulated genes, 17 were associative, forming protein-protein interaction network (**Figure 4A**). While 10 of the 100 down-regulated genes were also associative into protein-protein interaction network (**Figure 4B**).

To elucidate the functional enrichment of the associative DEGs obtained by STRING, we further performed KEGG enrichment analysis. The results revealed that, the 17 up-regulated associative DEGs were engaged in several key pathways, including RIG-I-like receptor signaling pathway, Hepatitis B, Viral carcinogenesis, Extrinsic apoptotic signaling pathway *via* death domain receptors, and innate immune response pathways, etc. (**Supplementary Table S13**). In contrast, the 10 down-regulated associative DEGs were involved in Focal adhesion, Amoebiasis, and Viral carcinogenesis pathways (**Supplementary Table S14**). These results indicate that the up-regulated DEGs in small intestine epithelial cells in non-adhesive group are associated with innate immunity and coupled with the lack of F4R, they could provide immunity against ETEC-F4ac induced diarrhea. While, the involvement of the down-regulated DEGs in Focal adhesion and Amoebiasis, this could imply that, their expression is linked to the presence of the F4R phenotype in small intestine epithelial cells in the adhesive group.

### Validation of the DEGs

To confirm the reproducibility of our RNA-Seq DEGs, we performed quantitative real time PCR (RT-qPCR). A total of 16 DEGs were selected for RT-qPCR validation, which included eight up-regulated genes (*DDX3X, ARPC4, CXCL2, CYCS, CTLA4, IL1R2, MGAT2*, *RBP2*, and *SLC22A8*) and eight down-regulated genes *(RUNX1T1, ACTN2, CACNB2, CLDN11*, *DGKG, HCN4, NCAM1*, and *MYLK*). It showed that the relative expression and fold change ratios of the 16 genes obtained from RT-qPCR were in coherent with the results of RNA-Seq data, confirming the reproducibility and reliability of our RNA-Seq data (**Figures 4C, D**).

## Discussion

Diarrhea episodes induced by ETEC F4ac in swine industries, hitherto persistent and very difficult to be averted or eradicated. This study investigated the transcriptome profiling of ETEC-F4ac non-adhesive vs. adhesive small intestine epithelial cells in Large White piglets using next generation sequencing technology. Our study focused on major genes and important pathways associated with F4R and susceptibility to ETEC-F4ac in small intestine epithelial cells of Large white piglets. Our results found a total of 543 DEGs *(P* < 0.05 and log_2_|FC| > 1) in comparison of ETEC-F4ac non-adhesive vs. adhesive small intestine epithelial cells (**Figure 1E**) and a total of 822 DEGs with *P < 0.05* (**Supplementary Table S3**). A previous study profiled the transcriptome of IPEC-J2 cell monolayers found 810 DEGs (FDR < 0.05) (Mirhashemi et al., 2018).

To identify major genes that are involved in important biological processes and pathways related to ETEC-F4ac adhesion, GO, and KEGG of genes exclusively expressed in non-adhesive and adhesive small intestine epithelial cells and DEGs in the comparison between the two groups were performed. Our study detected two (*SLC25A31, CNGA4*) (**Figure 2C**) exclusively expressed in adhesive small intestine cells involved in important pathways related to ETEC-F4 susceptibility. The *CNGA4* and *SLC2531* were engage in cAMP and cGMP-PKG signaling pathway respectively (**Supplementary Table S6**).

**Figure 5:**
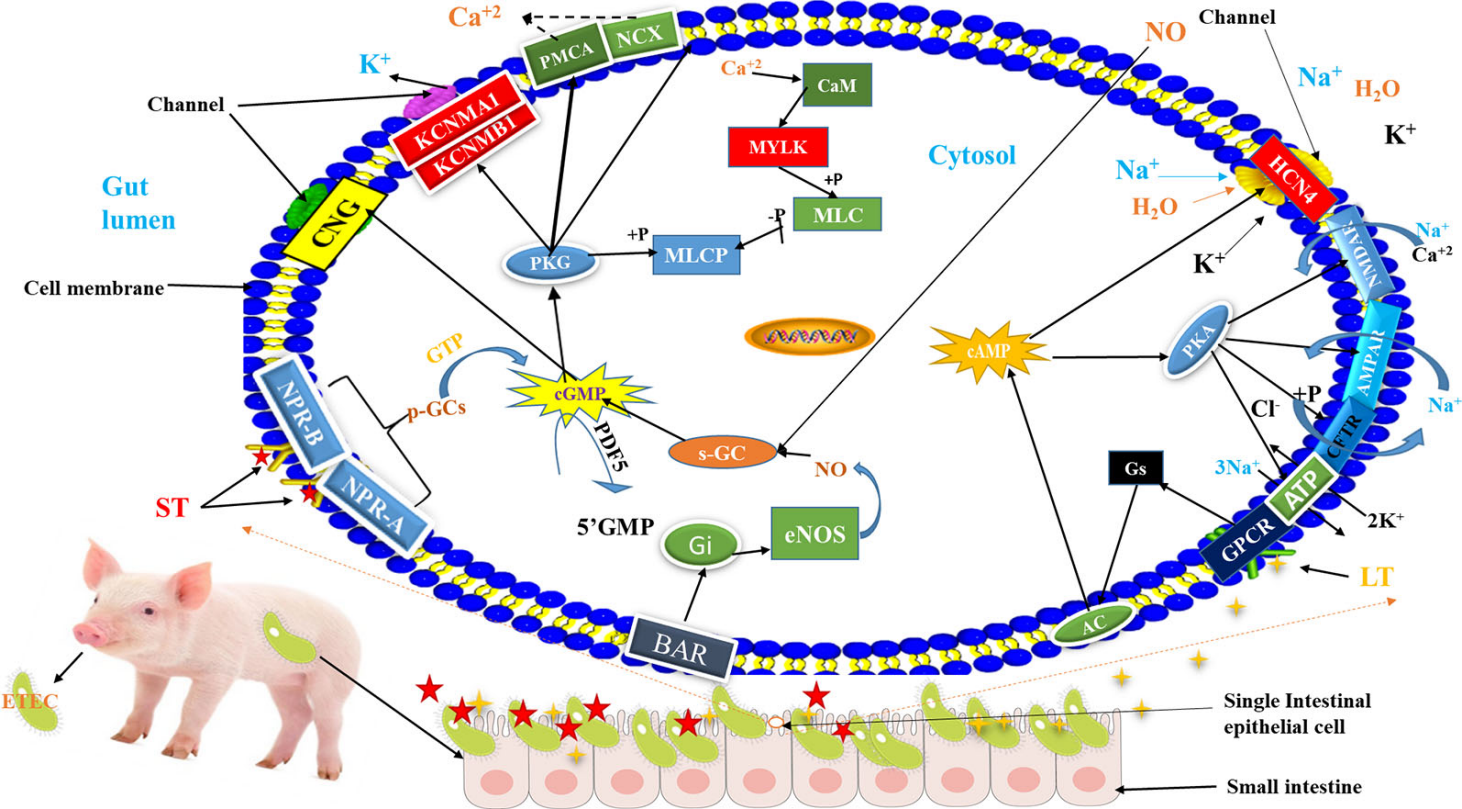
Schematic diagram of the mechanism of ETEC-F4ac diarrhea pathogenesis in swine: Briefly, after consumption of ETEC-F4ac through food or water, the bacteria reach small intestine of piglets and bind with their F4ac fimbriae to F4R on the surface of intestinal epithelial cells and establish colonization. Subsequently, they secret enterotoxins called heat labile (LT) (Fasano, 2008) and heat stable (ST) (Romi et al., 2012). Then, G protein-coupled receptor at the cell's surface membrane receives the LT toxin and passes it to GNAs complex locus (Gs) which activates adenylate cyclase (AC) (Greene et al., 2013), and natriuretic peptide receptor 1 (NPR-A/NPR-B) receives ST which activates soluble guanylyl cyclase (p-GCs) (Romi et al., 2012). The AC catalases the cyclization of Adenosine monophosphate (AMP) to generate cyclic adenosine monophosphate (cAMP) inside the cell (Greene et al., 2013), whereas guanylyl cyclase (p-GCs), catalyzes the conversion of GTP into cGMP to activate protein kinases (PKG) (Romi et al., 2012). Increased intracellular cAMP interferes with the function of Hyperpolarization activated cyclic nucleotide gated potassium channel 4 (*HCN4)* that controls the movement of electrolytes in and out of the cell, thus leaving the gate (channel) opened allowing more influx of water and electrolytes in to the gut lumen causing diarrhea (Greene et al., 2013). While, the activation of p-GCs increases intercellular cGMP, which in turn, has direct effect on cGMP-dependent protein kinases (PKG) (Hofmann et al., 2006), that interferes with the function of Potassium calcium-activated channel M regulatory beta subunit 1/alfa subunit 1 (*KCNMB1/KCNMA1*), leading to more secretions of electrolytes into gut lumen causing diarrhea. For more details, please, refer to **Supplementary Figures 2** and **3**.

On the other hand, we also detected the down-regulation of *HCN4, MYLK, KCNMB1* and *KCNBA1* in the comparison of non-adhesive vs. adhesive small intestine epithelial cells involved in important pathways related to ETEC-F4ac susceptibility. The *HCN4* was involved in cAMP signaling pathway and *MYLK, KCNMB1*, and *KCNMA1* were engaged in cGMP-PKG pathway (**Table 3** and **Supplementary Table S14**). *KCNMA1* is listed in top 25 DEGs (**Figure 1F**), which is up-regulated in adhesive group. The up-regulation of these genes might be due to a functional F4R in adhesive group in addition to TT genotype at *ITBG5* SNP that has been identified by (Fu et al., 2012) as a promising candidate gene underlying susceptibility to both ETEC-F4ab/ac. This is because ETEC-F4ac strain that colonizes small intestine, produces heat stable (ST) and heat labile (LT) toxins (Fasano, 2008; Hodges and Gill, 2010). These toxins activate Guanylyl cyclase (GC) and Adenylyl cyclase (AC), respectively. The GC catalases the cyclization of guanine monophosphate to cyclic guanine monophosphate (cGMP) (Greene et al., 2013; June, 2017). Whilst AC enzyme catalases the cyclization of adenine monophosphate forming cyclic adenine monophosphate (cAMP) (Sassone-Corsi, 2012) (**Figure 5**). Increasing cGMP and cAMP levels in the cell causes hypersecretion of fluids and ions into gut lumen (Romi et al., 2012; Read et al., 2014) thus leading to watery diarrhea.

In addition, our results also detected the involvement of up-regulated DEGs in several immune response-related pathways (**Supplementary Tables S7** and **S13**). Their expression may be linked to the lack of a functional F4R preventing adhesion of ETEC-F4ac to small intestine epithelial cells.

Intriguingly, neither of these pathways was enriched for the down-regulated DEGs. The results provide insights for the phenotypic differences between the two groups with regard to presence and absence of a functional F4R. The differences could also reflect differences in the gut microbiome of the pigs, the breeding value of the animals for adhesion phenotype, or other factors not explored in this study. We will therefore seek to address them in future studies.

The activation of MAPK signaling pathways by the down-regulated DEGs revealed in this study could have resulted in intestinal dysfunction (Syono and Ito, 1978), which might have led to the manifestation of adhesive phenotype observed in small intestine epithelial cells.

Furthermore, the involvement of up-regulated *IL7 a*nd *IL1R2* in cytokine-cytokine receptor interaction pathway (**Supplementary Table S13**) revealed in this study, may be involved in the induction of the inflammatory response against ETEC-F4ac infection. And thus non-adhesive piglets acquired adaptive immunity as a result. This is because, cytokine-receptor interaction may be critical in discovering the effects of inflammation in the disease development (Qian et al., 2019). The production of cytokines may lead to the activation of leukocytes and elimination of the infective agents (Zhou et al., 2017).

Epithelial cells have been reported in many studies exhibiting expression of both chemokines and chemokine receptors (Brand et al., 2006; Brand et al., 2006) with differentially up-regulated in uninfected intestine (Diegelmann et al., 2011). The ability of epithelial cells in intestine to secrete both cytokines and chemokines in response to pathogens or injury permits them to play critical role in shaping the nature of the local immune response (Trivedi and Adams, 2018). This is in coherent with the current study, which found *CXCL2* (chemokine) gene (among the top 25 DEGs in **Figure 1F**) with up-regulation pattern in non-adhesive small intestine epithelial cells (**Figure 3C**).

Further evidence to prove this, is the involvement of *CXCL2* in Chemokine signaling pathways, TNF signaling pathway, Salmonella infection, and Legionellosis pathways (**Supplementary Table S13**), suggesting that this gene could play a role in the immunity and its up-regulation reported in this study may be due to the absence of F4R in non-adhesive small intestine epithelial cells.

Moreover, the expression of *CTLA4* was up-regulated in the comparison of non-adhesive (non-infected) and adhesive (infected) small intestine epithelial cells, which is in consistent with many previous studies in humans and animals (Walker and Sansom, 2015; Khailaie et al., 2018; Xiang et al., 2018). The involvement of *CTLA4* (**Supplementary Table S13**) in cell adhesion and T cell receptor signaling pathway might also play positive role in immune related-response (Manley, 2013). A review by Lucy (Walker, 2013) entitled “Treg and *CTLA-4*: Two intertwining pathways to immune tolerance” reported that both regulatory T cells (Treg) and the *CTLA-4* pathway are crucial for the control of homeostasis in immunity, we believe it could also control fluid homeostasis in small intestine epithelial cells in non-adhesive group and therefore prevent over secretion of fluids into gut lumen. In addition, as an immune tolerance critical regulator, the loss of expression of *CTLA-4* in animal models is lethal shortly post-birth owing to extensive proliferation of lymphocytes and autoimmunity (Walker, 2017; Hou et al., 2018).

Intriguingly, we detected DDX3X with up-regulation pattern in small intestine epithelial cells in non-adhesive group is engage into all significantly enriched biological processes. This is an indication that, this gene may play a role in innate immunity. This is in coherent with Chen et al., 2014 who reported that, *DDX3X* involved in the innate immune signaling pathways leading to induced type I interferon in porcine. In addition to this, they also discovered that porcine *DDX3X* has an antiviral effect during PRRSV infection (Chen et al., 2014).

Finally, the up-regulation of DEGs in non-adhesive small intestine epithelial cells revealed by this study whether as responses to microbial flora in the gut or due to innate immunity is unclear. It thus, warrants further investigation. In addition, considering the limited sample size in the current study (four non-adhesive pigs vs. four adhesive pigs), although RNA-seq revealed many genes exclusively and differentially expressed in the comparison of non-adhesive vs. adhesive small intestine epithelial cells, these genes require validation in a larger pig population in future studies.

In conclusion, our study identified many genes and pathways associated with innate immunity and susceptibility to diarrheal infection caused by ETEC-F4ac. Six up-regulated DEGs (*CYCS, IL7, IL1R2, CXCL2*, *CTLA4*, and *DDX3X)* were found involved in many immune related pathways. Two genes (*SLC25A31, CNGA4*) exclusively expressed in adhesive small intestine cells and four (*HCN4*, *MYLK, KCNMB1*, and *KCNMA1)* DEGs with up regulation pattern in adhesive small intestine epithelial cells as a result of a functional F4R. These genes *via* their direct involvement in cAMP and cGMP-PKG signaling pathways may play a role in ETEC-F4ac susceptibility phenotype, i.e., the consequences of F4 being able to bind to the epithelium and produce toxin. One gene (*MYLK*) out of the six genes is located on SSC13 locus near to a 2.3 Mb region for F4acR described by (Ren et al., 2012), this strongly support that it might play a role in the adhesion phenotype of ETEC-F4ac to F4R in the small intestine epithelial cells in adhesive group, but need further follow up validation using association studies and linkage analysis.

Generally, the DEGs observed in this study may be inextricably linked with the differences in F4R phenotypes and SNP (C > T) genotypes at *ITGB5* between non-adhesive and adhesive small intestine epithelial cells.

Consequently, these 12 genes could be considered as candidate genes whose expression pattern is inextricably linked to absence and presence of F4R in small intestine epithelial cells.

## Data Availability Statement

The sequencing data associated with this study has been uploaded to NCBI and can be found using the following accession numbers:

Sequence Read Archive (SRA) submission: SUB6230222. BioProject accession: PRJNA562774.
8 SRRs: (SRR10037916 –to- SRR10037923).
BioSample accession: SAMN12649810.

## Ethics Statement

The animal study was reviewed and approved by Animal Welfare Committee of China Agricultural University (Permit Number: DK996).

## Author Contributions

QZ contributed conception of the study. YY designed and supervised the study. SA conducted all the experiments and wrote the manuscript. QX helped in conducting some experiments, gathered literature, and organized data. XL analyzed RNA-Seq data. LL helped in data analysis and interpretation. All authors contributed to manuscript revision, read, and approved the submitted version.

## Funding

This research was financially supported by the National Natural Science Foundation (NSFC31572361), the program for Chang Jiang Scholar and Innovation Research Team in University (IRT-15R62).

## Conflict of Interest

The authors declare that the research was conducted in the absence of any commercial or financial relationships that could be construed as a potential conflict of interest.
