# Supplementary figures and images for: Transcriptomic Study of Porcine Small Intestine Epithelial Cells Reveals Important Genes and Pathways Associated With Susceptibility to Escherichia coli F4ac Diarrhea
Source: Front Genet. 2020 Feb 27;11:68. doi: 10.3389/fgene.2020.00068 (PMC7056726; doi:10.3389/fgene.2020.00068)

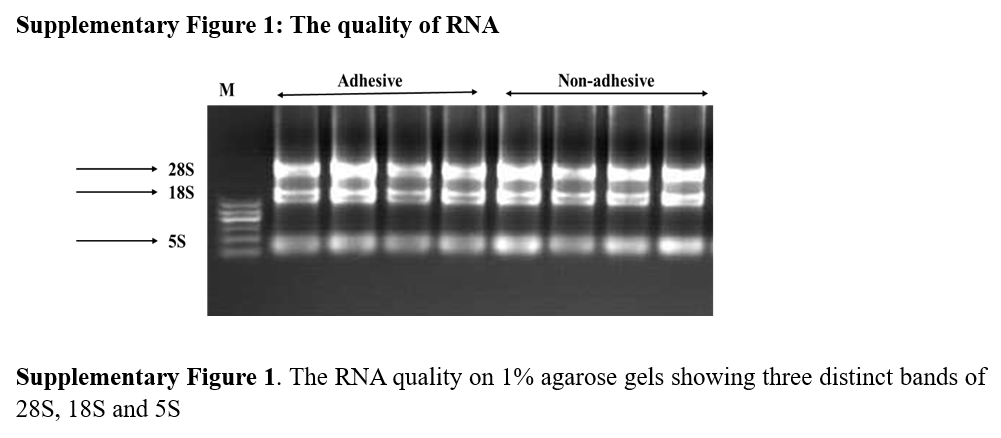

Supplement: Supplementary file 1 [file Image_1.tif]

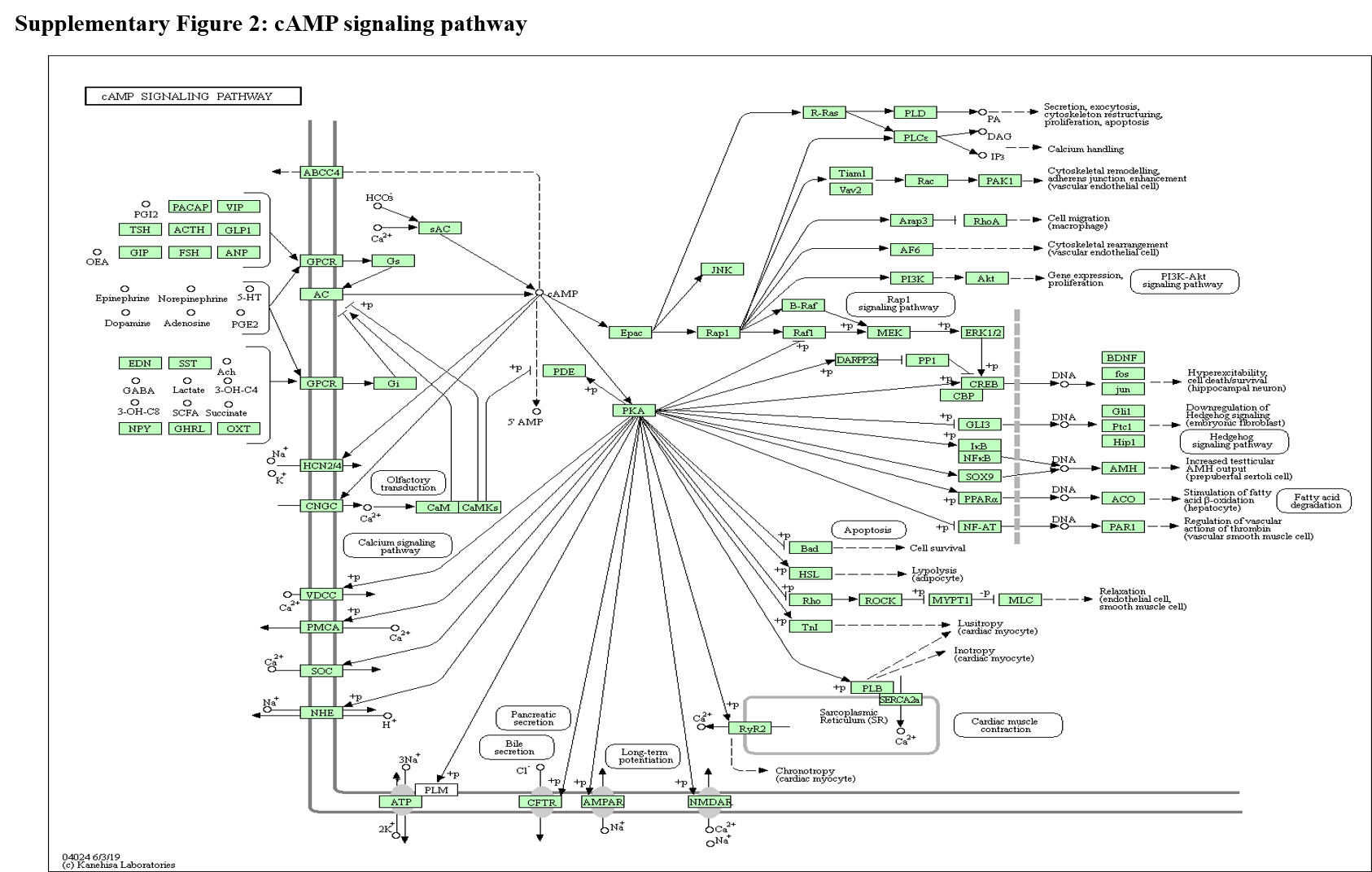

Supplement: Supplementary file 2 [file Image_2.tif]

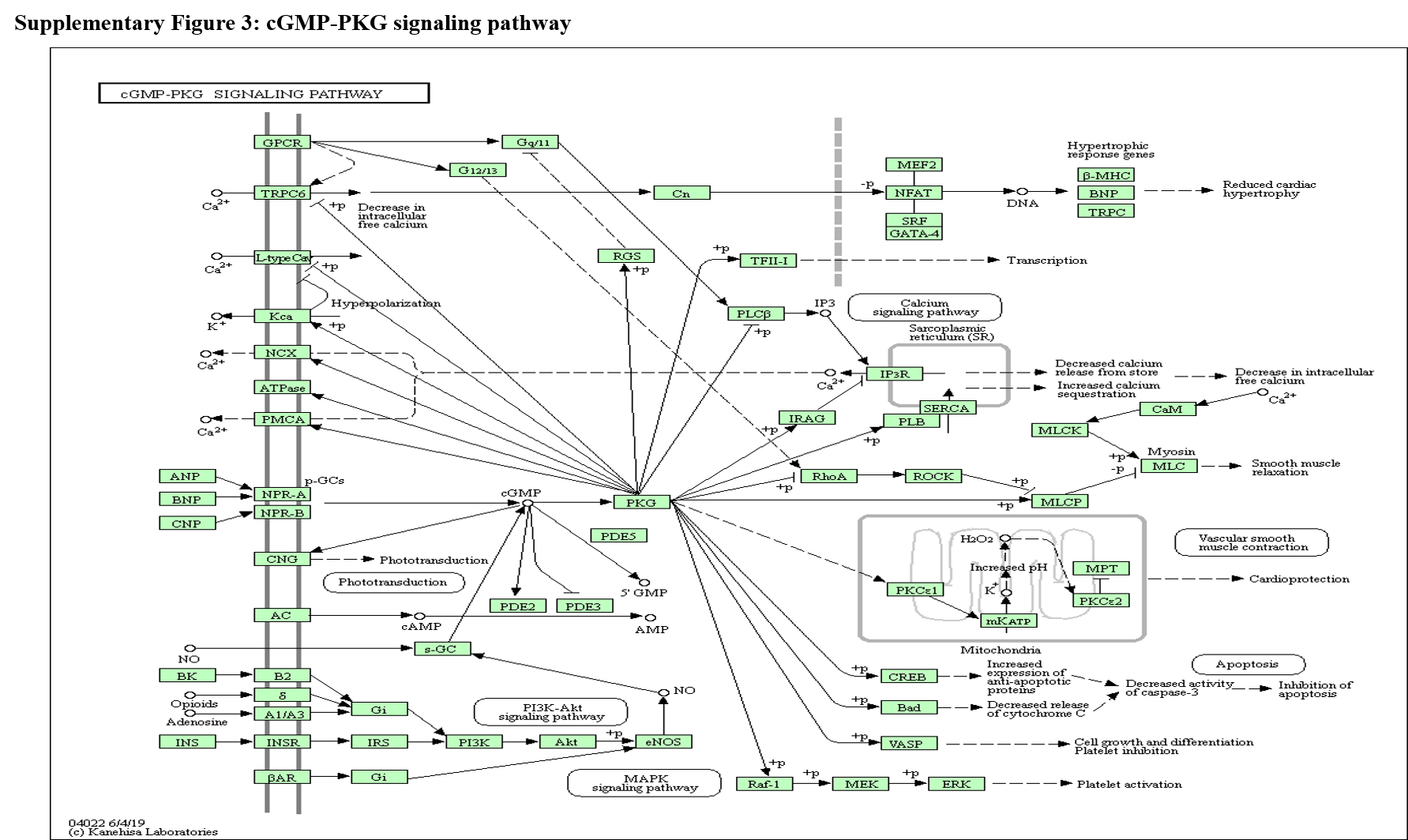

Supplement: Supplementary file 3 [file Image_3.tif]
