# Supplementary material for: Transcriptomic Study of Porcine Small Intestine Epithelial Cells Reveals Important Genes and Pathways Associated With Susceptibility to Escherichia coli F4ac Diarrhea
Source: Front Genet. 2020 Feb 27;11:68. doi: 10.3389/fgene.2020.00068 (PMC7056726; doi:10.3389/fgene.2020.00068)
Supplement: Supplementary file 4 [file Table_1.docx]

**Supplementary table S1. Number of raw reads, clean reads and percentage of clean reads, assigned alignments and genes in each sample**

|  |  |  | **Non-adhesive group** | | |  |  | |  | | | **Adhesive group** | | |  |  |
| --- | --- | --- | --- | --- | --- | --- | --- | --- | --- | --- | --- | --- | --- | --- | --- | --- |
| **Sample** | **1** | | | **2** | **3** | **4** | |  | | **1** | **2** | | **3** | **4** | | |
| **Raw Reads** | **123574890** | | | **115189226** | **127370354** | **122342616** | |  | | **126569978** | **130924082** | | **126760832** | **126165252** | | |
| **Clean Reads** | **121798254** | | | **113294846** | **125627966** | **120851962** | |  | | **124768650** | **129340552** | | **124748322** | **124240216** | | |
| **Clean Reads (%)** | **98.56** | | | **98.36** | **98.63** | **98.78** | |  | | **98.58** | **98.79** | | **98.41** | **98.47** | | |
| **Assigned alignments** | **82289709** | | | **72634243** | **75692862** | **80018318** | |  | | **67923106** | **76193802** | | **80080616** | **78535281** | | |
| **Genes** | **17050** | | | **13278** | **15946** | **18233** | |  | | **17684** | **17873** | | **17379** | **17966** | | |
