# Supplementary material for: Transcriptomic Study of Porcine Small Intestine Epithelial Cells Reveals Important Genes and Pathways Associated With Susceptibility to Escherichia coli F4ac Diarrhea
Source: Front Genet. 2020 Feb 27;11:68. doi: 10.3389/fgene.2020.00068 (PMC7056726; doi:10.3389/fgene.2020.00068)
Supplement: Supplementary file 7 [file Table_4.docx]

**Supplementary Table S4: Significantly enriched KEGG pathway for genes exclusively expressed in epithelial cell in non-adhesion group**

|  |  |  |  |
| --- | --- | --- | --- |
| KEGG pathway | KEGG ID | *Genes* | P value |
| MicroRNAs in cancer | ssc05206 | *ssc-mir-331, MIR335, ssc-mir-143, MIRLET7G, ssc-mir-181b-1, ssc-mir-23b, MIR199B, MIRLET7I, ssc-mir-101-2, IGF2BP1* | 2.32E-05 |
