# Supplementary material for: Transcriptomic Study of Porcine Small Intestine Epithelial Cells Reveals Important Genes and Pathways Associated With Susceptibility to Escherichia coli F4ac Diarrhea
Source: Front Genet. 2020 Feb 27;11:68. doi: 10.3389/fgene.2020.00068 (PMC7056726; doi:10.3389/fgene.2020.00068)
Supplement: Supplementary file 8 [file Table_5.docx]

**Supplementary Table S5: Significantly enriched KEGG pathways for genes exclusively expressed in epithelial cells in adhesion group**

|  |  |  |  |
| --- | --- | --- | --- |
| **KEGG pathway** | **KEGG ID** | ***Genes*** | **P value** |
| Olfactory transduction | ssc04740 | *CNGA4, OR6K6, OR51F1, OR6A2, OR51E2, OR51D1, ENSSSCG00000023516, ENSSSCG00000005801, ENSSSCG00000006411, ENSSSCG00000013851, ENSSSCG00000012665, ENSSSCG00000007959, ENSSSCG00000014756, ENSSSCG00000014755, ENSSSCG00000014704, ENSSSCG00000013123, ENSSSCG00000021523, ENSSSCG00000005419, ENSSSCG00000026768, ENSSSCG00000029107, ENSSSCG00000028929, ENSSSCG00000030554, ENSSSCG00000030537* | 0.002 |
| MicroRNAs in cancer | ssc05206 | *ssc-let-7c, MIR92A-1, ssc-mir-103-1, ssc-mir-423, MIR10A, ssc-mir-18a, ssc-mir-181a-1, ssc-mir-16-2, ssc-mir-103-2, ssc-mir-7-2* | 0.003 |
| Steroid hormone biosynthesis | ssc00140 | *CYP7A1, CYP19A1, ENSSSCG00000010546, ENSSSCG00000026798, ENSSSCG00000008935* | 0.004 |
| Drug metabolism - cytochrome P450 | ssc00982 | *ALDH3A1, ENSSSCG00000006320, ENSSSCG00000022759, ENSSSCG00000008935* | 0.025 |
| Chemical carcinogenesis | ssc05204 | *ALDH3A1ENSSSCG00000010546, ENSSSCG00000026798, ENSSSCG00000008935* | 0.042 |
| Arachidonic acid metabolism | ssc00590 | *GPX5, ALOX15B, ENSSSCG00000010546, ENSSSCG00000026798* | 0.045 |
